# Advancements and challenges in amyotrophic lateral sclerosis

**DOI:** 10.3389/fnins.2024.1401706

**Published:** 2024-05-22

**Authors:** David Bradford, Kathleen E. Rodgers

**Affiliations:** Department of Medical Pharmacology, Center for Innovation in Brain Science, University of Arizona College of Medicine, Tucson, AZ, United States

**Keywords:** amyotrophic lateral sclerosis, inflammation, excitotoxicity, metabolism, neurodegenerative disease

## Abstract

Amyotrophic lateral sclerosis (ALS) continues to pose a significant challenge due to the disease complexity and heterogeneous manifestations. Despite recent drug approvals, there remains a critical need for the development of more effective therapies. This review explores the underlying mechanisms involved; including neuroinflammation, glutamate mediated excitotoxicity, mitochondrial dysfunction, and hypermetabolism, and how researchers are trying to develop novel drugs to target these pathways. While progress has been made, the unmet need of ALS patients highlights the urgency for continued research and resource allocation in the pursuit of effective treatments.

## 1 Introduction

Amyotrophic lateral sclerosis (ALS) is a rare, progressive neurodegenerative disease leading to a rapid loss of motor neurons, leading to complete paralysis and death. Classified as an orphan disease, the prevalence for ALS is as high as 9.9 per 100,000 people, equating to about 31,843 people nationally (Mehta P. et al., 2023). Globally, this number increased to 222,801 in 2015, with this number expected to increase by 69% by 2040, highlighting the importance in understanding this devastating disease (Arthur et al., 2016). First identified in 1869 by Jean-Martin Charcot, ALS remained relatively unknown until 1941 when Lou Gehrig was diagnosed, and the term Lou Gehrig’s Disease was coined (The ALS Association, 2024a). It then took another 50 years for the first genetic risk factor, *SOD1* mutation, to be discovered (Rosen et al., 1993).

Beginning from symptomatic onset, the average life expectancy is between 2 to 5 years, with about half of people dying within 3 years (Štětkářová and Ehler, 2021; The ALS Association, 2024b). A challenge with treating this disease lays with the difficulties around diagnosing ALS. It can take up to 14 months between the first clinic visit to an accurate diagnosis, with some groups reporting a delay of up to 24 months at the high end (Chiò, 1999; Segura et al., 2023). This delay in diagnosis is detrimental to the patient’s overall treatment, causing a heavy burden to both the patient’s mental health and more importantly, drastically delaying targeted treatment. Unlike other diseases, ALS is difficult to diagnosis due to a number of confounding factors. Clinicians undergo a “rule-out” approach when diagnosing a patient. In fact, over half of patients receive a different diagnosis prior to the correct ALS diagnosis, with the four most common alternative diagnosis being neuropathy (28%), spinal disease (18%), vascular disease (11%), or another neurodegenerative disease (11%) (Paganoni et al., 2014). This difficulty in diagnosing is in part due to the heterogeneity of the disease. ALS can mimic a variety of different neurological and motor diseases. The symptoms a patient has comes from where the disease first manifests. The two main classifications are bulbar-onset or limb-onset. Bulbar-onset first arise in the head and neck, affecting speech and swallowing. Limb-onset first affect the muscles in the periphery, primarily the hands and feet (Masrori and Van Damme, 2020). To further complicate things, the majority of ALS cases are sporadic in nature, making up 90% of cases, where the remaining 10% are familial, having a genetic component (Brotman et al., 2023). Understanding the complexity of this neurodegenerative disease may serve as a starting point to stratify this heterogeneous population to better develop targeted therapies and find potential biomarkers for earlier diagnosis.

## 2 ALS risk factors

### 2.1 Genetic factors

To date there are over 50 genes implicated in the development of ALS affecting a variety of neuronal processes (Mejzini et al., 2019). The four most common mutations seen in fALS patients lay within mutations in the *SOD1, C9orf72, TARDBP, and FUS* genes, however there are multiple other genes with a definitive or likely causative link to ALS (Boylan, 2015). In addition to the previously mentioned genes with a definitive link to ALS, *kinesin family member 5A* (*KIF5A*), is also linked (Nicolas et al., 2018). KIF5, expressed in motor neurons, is responsible for cargo binding to adaptor proteins (Kanai et al., 2000). In ALS, there is a loss of function, disrupting axonal transport leading to neurodegeneration (Millecamps and Julien, 2013). In addition to these genetic mutations with a definitive link to ALS development, over 30 additional genes have been associated with a “likely causative” link. These include mutations in genes linked to the formation of toxic aggregates (*ANXA11, DCTN1, EWSR1, MATR3, NEFH, PRPH*, *TAF15, TIA1, UBQLN2,* and *VAPB*) or affecting mitochondrial function (*CHCHD10, CHMP2B, SIGMAR1, SQSTM1,* and *VCP*) (Smukowski et al., 2022). The wide variety of genes contributing to the development of ALS highlights the complexity of this disease and contributes to the challenges with treating this disease.

#### 2.1.1 *SOD1* mutations

The superoxide dismutase 1 (*SOD1*) gene has been a major focus of ALS research since it was first identified in 1993, making it the first genetic risk factor for familial ALS (fALS). *SOD1* encodes an antioxidant enzyme reducing oxidative stress by converting superoxide radicals into less reactive peroxide and oxygen (McCord and Fridovich, 1969). Despite being linked to a relatively small proportion of ALS cases (around 8–23% of all fALS cases and up to about 5% of all sporadic forms), the SOD1 mutations have provided crucial insights in the understanding of ALS pathology (Müller et al., 2022).

To date, over 200 unique mutations in the *SOD1* gene have been identified (Gagliardi et al., 2023). These mutations typically exhibit an autosomal dominant inheritance pattern, where only a single mutated copy of the gene is sufficient to increase susceptibility to ALS (Boylan, 2015; Feneberg et al., 2020). In the context of disease progression, mutated SOD1 works in a gain-of-function manner, evident by a myriad of studies knocking down or entirely knocking out SOD1 and seeing either a slowing in disease progression or no ALS like phenotype, respectfully (Reaume et al., 1996; Sau et al., 2007; Bunton-Stasyshyn et al., 2015). Largely cytosolic, SOD1 is also found within the mitochondrial inner membrane space, making these areas affected (Sturtz et al., 2001). The gain-of-function is presumably believed to a result of increased aggregation, destabilization of dimers, and oligomerization of the mutant SOD1 protein (Pansarasa et al., 2018). The formation of insoluble aggregates is also believed to go on and either be neurotoxic in nature or block normal intracellular signaling and cellular function (McAlary et al., 2016; Pansarasa et al., 2018). These aggregates can then go on and affect neighboring cells, leading to the progressive nature seen in ALS pathology. While there is clearly a role SOD1 plays in ALS, the question of their exact role and how these aggregates interact in cellular transmission remains areas of active investigation. Answering the questions around SOD1 mutations could be a future therapeutic avenue for future drug development.

#### 2.1.2 *C9orf* mutations

The *C9orf72* gene, located on the short arm of chromosome 9, gained prominence in ALS research due to its association with a hexanucleotide repeat expansion (GGGGCC), making it the most prevalent genetic mutation in both familial and sporadic forms of ALS. This repeat expansion is seen in upwards to 50% of all fALS cases and 10% of sALS (Umoh et al., 2016). First identified in 2011 as a link between ALS and Frontotemporal dementia (FTD), it is believed these repeats work again in a gain-of-function fashion (DeJesus-Hernandez et al., 2011; Renton et al., 2011). The normal function of *C9orf72* is still not fully understood, however it is thought to play a role in various cellular processes, including vesicle trafficking and autophagy (Sellier et al., 2016; Smeyers et al., 2021).

These repeats are often present in healthy individuals, but evidence shows that deleterious events may begin when the hexanucleotide repeat expansions reaches over 30 times (Zhang et al., 2015). In fact, most ALS patients with the *C9orf72* mutation have expansions that range from hundreds to thousands of repeats, far exceeding what is seen in healthy populations (Mejzini et al., 2019). The exact molecular mechanism underlying the toxicity that leads to ALS pathology remains up for debate and not completely understood. It is theorized that the G rich regions have a tendency to form highly stable quadruplex structures (G-quadruplex). These G-quadruplexes can go on and interfere with various cellular processes, interfering and improperly interacting with intracellular proteins, affecting telomere stability and RNA transcription, splicing, translation, and transport (Fratta et al., 2012; Endoh and Sugimoto, 2016). Patients with the *C9orf72* mutation often exhibit a variety of symptoms, including both motor and cognitive impairments, as evident by the overlap with FTD. Further research may provide insights into the toxic mechanisms these aggregates play, and may pave the way for targeted interventions that not only help alleviate ALS pathology, but also relieve FTD symptoms.

#### 2.1.3 *TARDBP* mutations

Another genetic mutation leading to the formation of ALS lies within the *TARDBP* gene, which encodes the TDP43 protein (Jo et al., 2020). Since its causative link was first discovered in 2008, more than 60 *TARDBP* mutations have been identified (Chen et al., 2021). In fact, an overwhelming number of ALS cases exhibit TDP-43 proteopathy. Notably, 97% of all ALS cases have TDP-43 aggregates present, regardless of disease classification (sALS vs. fALS), making this a pathological hallmark of ALS (Swarup et al., 2011; Ling et al., 2013).

TDP-43 is a versatile protein, involved in various cellular functions relating to RNA metabolism (Prasad et al., 2019). Under normal conditions, TDP-43 is primarily localized within the nucleus, contributing to the regulation of RNA processes and repressing the cryptic exon inclusion during RNA splicing (Jo et al., 2020; Ma et al., 2022). However, in ALS patients with TDP-43 proteopathy, there is a loss of functional TDP-43 within the nucleus and an increase in cytoplasmic levels (Arai et al., 2006). This loss of nuclear localization is a consequence from post-translational modifications, primarily through phosphorylation and ubiquitination (Neumann et al., 2006; Inukai et al., 2008). The accumulation of TDP-43 aggregates in the cytoplasm disrupts normal cellular processes, decreasing autophagy (by affecting TFEB [transcription factor EB] localization), increasing stress granule formation, and increases mitochondrial dysfunction (Liu-Yesucevitz et al., 2010; Stribl et al., 2014; Xia et al., 2016).

In addition to forming toxic aggregates, the loss of nuclear TDP-43 leads to a loss in RNA splicing regulation, often including cryptic exons leading to the inclusion of frameshifts or stop codons (Torres et al., 2018). In 2019, two independent groups found that the loss of functional TDP-43 leads to the inclusion of a cryptic exon within the *STMN2* gene, which encodes a microtubule-associated protein (stathmin-2) critical for axonal growth and neuronal repair (Klim et al., 2019; Melamed et al., 2019; Mehta P. R. et al., 2023). Other cryptic exons can be found implanted within genes due to the loss of TDP-43. For instance, these exons have been found in *ATG4B* (autophagy related 4B cysteine peptidase), inhibiting autophagy. The inclusion of cryptic exons have been primarily found within regions implicated in ALS, including the motor cortex and spinal cords of ALS patients (Ling et al., 2015; Torres et al., 2018). The role of TDP-43 seems to be two-fold, acting as both a gain-of-function (through their ability to form toxic aggregates) and a loss-of-function (evident by their mismanagement of RNA splicing). Even with this, the exact mechanisms through which TDP-43 aggregates contribute to neurodegeneration are actively under investigation with many groups looking at stabilizing their functions to alleviate ALS like symptoms. Inhibiting the formation of TDP-43 aggregates and promoting cytoplasmic clearance may be a promising target to treat ALS, not just in those with a *TARDBP* mutation.

#### 2.1.4 *FUS* mutations

*FUS* (Fused in Sarcoma) is a gene that encodes an RNA-binding protein crucial for various cellular processes, including transcription, translation, DNA repair, and the maintenance of genomic stability (Lagier-Tourenne et al., 2010; Chen et al., 2019). Mutations within the *FUS* gene are heavily linked to the development of ALS, especially in juvenile cases, where the *FUS* mutation is the most frequent genetic cause (Vance et al., 2009; Zou et al., 2016). The majority of disease-causing mutations are located at the C-terminal region, disrupting the nuclear localization signals (Nakaya and Maragkakis, 2018). These signals are responsible for mediating the transport of proteins from the cytoplasm into the nucleus (Lu et al., 2021). Instead of residing in the nucleus, the FUS is mislocalized into the cytoplasm, leading again to a gain of function phenotype, leading to neuronal toxicity and ALS pathology (Qiu et al., 2014). Like the other mutations mentioned, the specific mechanism of how *FUS* mutations lead to ALS is an ongoing area of research. However, it is clear that *FUS* mutations add to the complexity of this disease, further highlighting the heterogeneity present in ALS.

#### 2.1.5 Other risk and environmental factors

Outside of the genetic mutations, being a male is the strongest risk factor for developing ALS. Men have a 1.5 times greater risk of developing ALS than women. This is likely due to early testosterone exposure possibly driving the disease pathogenesis (Mehta et al., 2014). This trend continues up until menopause, where after menopause, the incidence rates between men and women equal out (Oskarsson et al., 2015). Even with this increase in prevalence, only one mutation is linked to the X chromosome, with the others acting in an autosomal dominant fashion (Benarroch et al., 2023). Therefore, this risk factor could also be tied to an increase in exposure to other risk factors described below.

Outside of the genetic risk factors, there are no definitive risk factors that are known to cause the development of ALS. However, many groups have investigated whether certain environmental factors lead to a higher incidence of disease. It is believed that military experience has an increased incidence compared to non-military personnel (Beard and Kamel, 2015). This could also be true due to exposure of other neurotoxic agents like lead, pesticides, and smoking (Armon, 2009; Malek et al., 2012; Wang et al., 2014; Nieh et al., 2021). Another explanation for this trend in military personnel is with their overall physical fitness. Having a low body mass index is also associated with an increased risk of ALS, however, the causative link remains unknown (Mattsson et al., 2012). While these trends exist in certain cohorts, it should be emphasized that these are not definitive, with further research being needed to find the causative link, if there is one.

## 3 ALS pathology

Since its discovery, ALS has been long time viewed as an isolated muscle disease; however, sentiment within the field has been changing as this disease has systemic effects not isolated to voluntary muscles (Silani et al., 2017). Regardless of the disease classification, there are shared mechanisms contributing the neurodegeneration. While the exact mutation leading to the onset of the disease vary; neuronal hyperexcitability, neuroinflammation, and mitochondrial dysfunction are seen throughout disease progression.

### 3.1 Neuroinflammation

Although not unique to neurodegenerative diseases, neuroinflammation and immune activation are seen throughout the disease course of ALS. Neuroinflammation, led by microglial and astrocyte activation, T-lymphocyte infiltration, and the overproduction of proinflammatory cytokines are observed before symptomatic onset, suggesting a link between the immune response and ALS progression (Komine and Yamanaka, 2015). The balance between the protective vs. toxic role of immune activation remains elusive. Microglia act as the first line of defense within the CNS, surveying the microenvironment existing in two states; resting or activated (Cherry et al., 2014). Once activated, microglia can either enter one of two opposing pathways, the classical (M1) phenotype or alternative (M2) phenotype (Franco and Fernández-Suárez, 2015; Thompson and Tsirka, 2017).

In the presence of an injury, microglia become classically activated; exhibiting a proinflammatory and cytotoxic phenotype (Orihuela et al., 2016). These microglia produce inflammatory cytokines and chemokines, including but not limited to tumor necrosis factor alpha (TNFα), interleukin (IL)-1b, IL-6, IL-12, and increased oxidative stress (Villalta et al., 2009; Colonna and Butovsky, 2017). TNFα has been shown to have a direct effect on neuronal excitability both *in-vivo* and within hippocampal slices from animals with peripheral inflammation (Riazi et al., 2008; Galic et al., 2012). Other proinflammatory cytokines and chemokines, like the interleukins, also lead to neuronal death via the NF-kB signaling pathway, ultimately leading to apoptosis and neuronal loss (Wang et al., 2015; Singh and Singh, 2020). Increasing oxidative stress and excessive formation of reactive oxygen species (ROS) exacerbate the neurodegenerative process in a feed-forward pathway. ROS, released from damaged neurons or glial cells, can bind to glutamate receptors, thus elevating synaptic glutamate furthering neuronal excitability. This causes intracellular calcium to increase, causing mitochondrial dysfunction and directly causing more ROS production (Heath and Shaw, 2002; Hemerková and Vališ, 2021).

Another integral glial cell involved in neuroinflammation are astrocytes. Like microglia, astrocytes can become polarized, acting in either a neuroprotective role or facilitating neurodegeneration and releasing neurotoxins (Li et al., 2019). Cytotoxic astrocytes upregulate proinflammatory cytokines (like IL-1 and TNFα), which promotes neuronal inflammation. These astrocytes also upregulate glutamate and ATP secretion (Orellana et al., 2011; Liddelow and Barres, 2017). This increase in glutamate release is also paired with a downregulation in excitatory amino acid transporter 2 (EAAT2) expression further promoting another neurodegenerative pathway, glutamate mediated excitability (Gong et al., 2000). These studies highlight the detrimental effects elevated proinflammatory agents has on organism as a whole, and not just locally where they are produced.

### 3.2 Hyperexcitability

Glutamate-mediated excitotoxicity has been widely observed within ALS. So much so that the first FDA approved medication for ALS, Riluzole, targets this phenomenon (Doble, 1996). Under normal physiological conditions, glutamate release and clearance are highly regulated. So much so that within CNS, 1% of the total brain proteins are the glial glutamate transporter, EAAT2 (Danbolt, 2001; Foran and Trotti, 2009). This highlights the importance of quickly clearing out released glutamate and preventing glutamate from spilling over to neighboring synapses.

Alterations in glutamate homeostasis in ALS pathology has been widely established, but the cause remains unknown. As alluded to earlier, ALS patients demonstrate both an increase in glutamate release as well as a decrease in glutamate uptake, primarily due to the loss of the astrocytic glutamate transporter, EAAT2 (Rothstein et al., 1992; Ferrarese et al., 2001). Incorrect splicing due to abnormal EAAT2 mRNA were found in ALS patients (Lin et al., 1998). This decrease in clearance leads to an accumulation of glutamate, leading to neurotoxic levels and further pathogenesis. The levels of glutamate are elevated in both the cerebrospinal fluid (CSF) and plasma, indicating compensatory mechanisms are unable to adapt, leading to systemic stress on the patient.

### 3.3 Mitochondrial dysfunction

The accumulation of damaged mitochondria is a prevalent feature in age-related neurodegeneration, and a common feature in ALS (Kim et al., 2019). As previously mentioned, neuronal inflammation and glutamate mediated excitotoxicity contribute to the development of mitochondrial dysfunction. Mitochondria are the main site for ATP synthesis and metabolism and their function is essential, especially in energy-demanding organs like the brain and muscle (Balasubramanian, 2021; Zhao et al., 2022). Therefore, mitochondrial dysfunction is heavily linked to ALS pathogenesis and disease progression. Prolonged elevated intracellular calcium overloads the mitochondria, resulting in ROS production, oxidative stress and eventually apoptosis (Kawamata and Manfredi, 2010).

In addition to calcium buffering, mitochondrial dysfunction leads to axonal transport dysfunction (Genin et al., 2023). ALS patients have mitochondrial with abnormal morphology, which includes increased cristae and mitochondrial swelling (Sasaki and Iwata, 2007). These alterations are all believed to affect ATP production and mitochondrial respiration. ALS mitochondria exhibit decreased activity of the electron transport chain (ETC), with defects seen across most aspects, including complex I, II, IV as well as certain mitochondrial enzymes, including citrate synthase and cytochrome c oxidase (Borthwick et al., 1999; Wiedemann et al., 2002; Vandoorne et al., 2018). Impaired cellular respiration and ATP production also contribute to a phenomenon typically seen in ALS patients, hypermetabolism.

### 3.4 Hypermetabolism

Hypermetabolism is a state of exhibiting an elevated resting energy expenditure (REE) and has been seen in upwards between 55–60% of ALS patients (Bouteloup et al., 2009; D'Amico et al., 2021). Other groups have found that the REE is increased by 10% on average in ALS patients when compared to that of healthy populations (Desport et al., 2001). In fact, ALS patients are recommended to consume more calories than their calculated need (Kasarskis et al., 1996; Ngo et al., 2014). It is not surprising that metabolic dysfunction would be implicated in ALS considering the brain and muscles are two of the most metabolically active tissue groups in the body (Wang et al., 2010).

Neurons and muscles, along with other tissues, utilize glucose as their main source of energy (Mergenthaler et al., 2013). Glucose is metabolized into pyruvate, via glycolysis, which can go on to produce more ATP by the TCA cycle. Under normal conditions, the energy required for homeostasis is met, promoting proper synaptic function and axonal transport in the neuron, two processes heavily reliant on glycolysis (Zala et al., 2013; Rangaraju et al., 2014). In both ALS patients and in SOD1 symptomatic mice, decreased levels of pyruvate have been observed, indicating an impairment of glucose utilization and the glycolytic pathway (Tefera et al., 2019, 2021).

With defects in the glycolytic pathway, the body must get energy from another source in order to meet the increased energy demand. One way the body does this is through b-oxidation, where lipids and fatty acids are utilized rather than glucose. While this process is normally fine short term, prolonged β-oxidation results in the formation of ROS and cellular stress (Zhang et al., 2010). When agents are added to promote glycolysis and the entry of pyruvate into the Krebs cycle, like dichloroacetate, to improve glycolytic capacity, there is a delay in muscle denervation and atrophy (Palamiuc et al., 2015). This switch from glycolysis to b-oxidation is shown to be cytotoxic in neurons and muscles, contributing to muscle denervation and disease progression.

**Figure 1:**
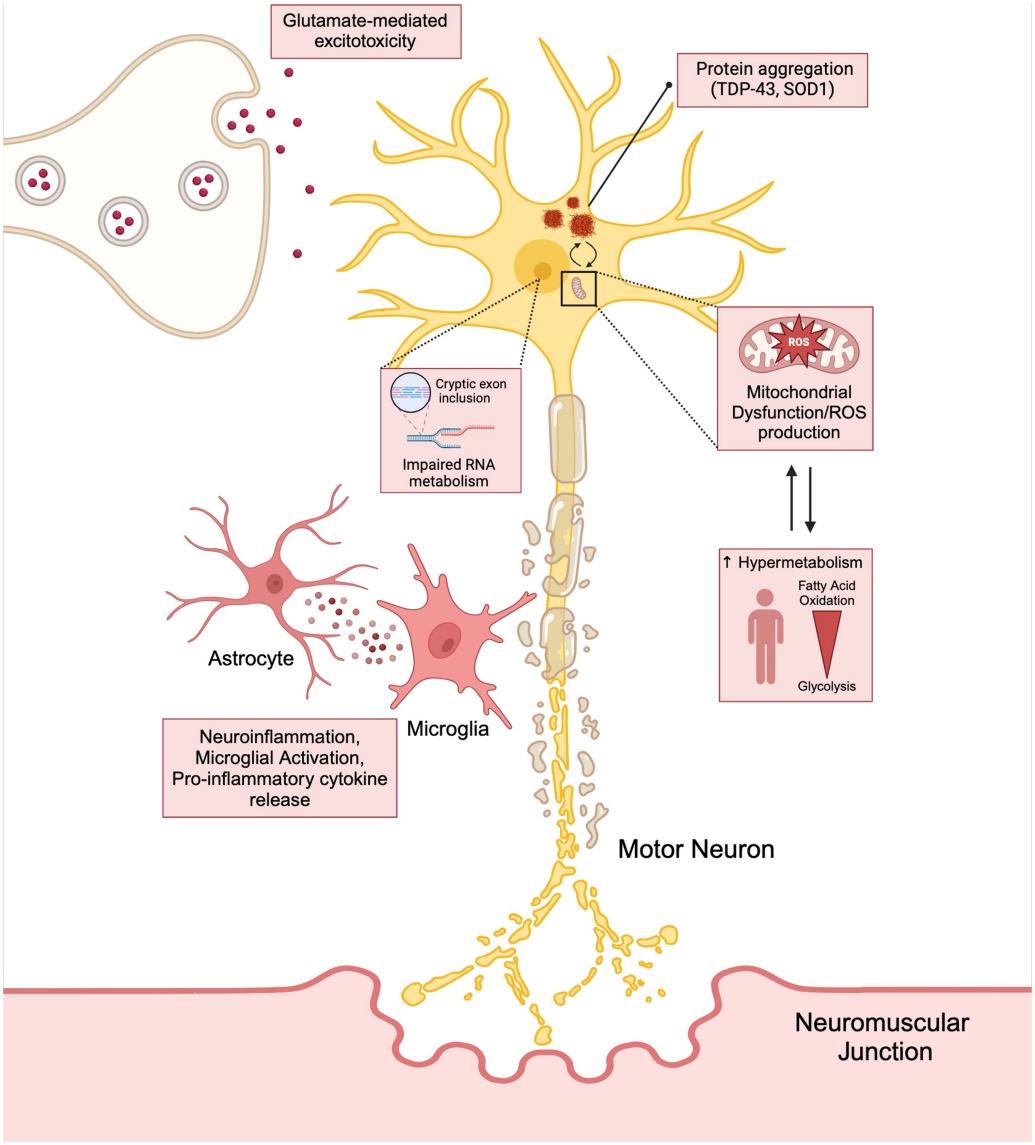
The interconnection between the hallmarks of ALS (neuroinflammation, glutamate mediated excitotoxicity, and mitochondrial dysfunction) have on stressing the motor neuron and how these cytotoxic pathways lead to disease progression and hypermetabolism. Created with BioRender.com.

It is important to note that these mechanisms leading to neurodegeneration and ALS disease progression should not be viewed as mutually exclusive, but rather a highly dynamic and reactive process potentially exacerbating one another (Figure 1). This phenomenon may be responsible for the failure in drug development and the limited benefit in the currently approved treatment options available for patients.

## 4 Drug discovery and clinical trial pipeline

Drug development is a long and complex process, often taking up to 15 years and costing over a billion dollars (Hughes et al., 2011). With the unmet need for viable new therapeutics, drug repurposing may be a more time and cost effect strategy. The utilization of network-based approaches, like SAveRunner, GEO, and STRING databases allow for the identification of possible targets with theoretical feasibility in ALS (Fiscon et al., 2021; Sunildutt et al., 2024). These methods have facilitated research in drugs with known safety profiles, speeding up clinical trial opportunities.

Many drugs undergoing clinical trials for ALS involve repurposed drugs that are already FDA approved for another indication. These include drugs previously approved for cancer treatments (Bosutinib and Mastinib), HIV treatments (Dolutegravir, Abacavir, and Lamivudine), and rheumatoid arthritis (Baricitinib) (Greig and Deeks, 2015; Isfort et al., 2018; Urits et al., 2020; Ketabforoush et al., 2023). Additional agents being investigated preclinically include autophagy inducers (Rapamycin), hormone antagonists (Tamoxifen and Imatinib), Alkylating agents (Cisplatin and Carboplatin), and immunomodulating drugs (Thalidomide and Lenalidomide) (Mandrioli et al., 2023; Potenza et al., 2024).

In recent years, there has been a push for accelerated approvals and drug development for rare diseases. The National Center for Advancing Translational Sciences (NCATS) plans to grow and improve the experimental drug pipeline for rare diseases (Mullard, 2023). Since the first drug was approved in 1995, three others have been approved, with two of those coming within the last two years (Saitoh and Takahashi, 2020; Center for Drug Evaluation and Research, 2023a,b). Even with these new treatment options for patients, there is still a desperate need to develop more effective therapies. This fact is especially evident by the recent determination of Relyvrio. Approved by the FDA in 2022, Relyvrio was taken off the market due to failed phase-III results, where there was no change in the disease progression in patients with or without treatment (Bryson, 2024). This recent withdrawal highlights the complexity and difficulties surrounding the development of effective treatment.

Currently, there are 35 clinical trials in phase I, II, or III that are recruiting on clinicaltrials.gov. Of these 35 trials, 29 are in either phase 1 or 2, with the primary focus of those drugs broadly targeting the immune system and neuroinflammation. Other targets involve either targeting neurons directly through the integrated stress response and oxidative stress, trying to mitigate symptoms and serving as comfort care, or looking at diagnostic approaches through imaging for screening and faster disease identification.

**Figure 2:**
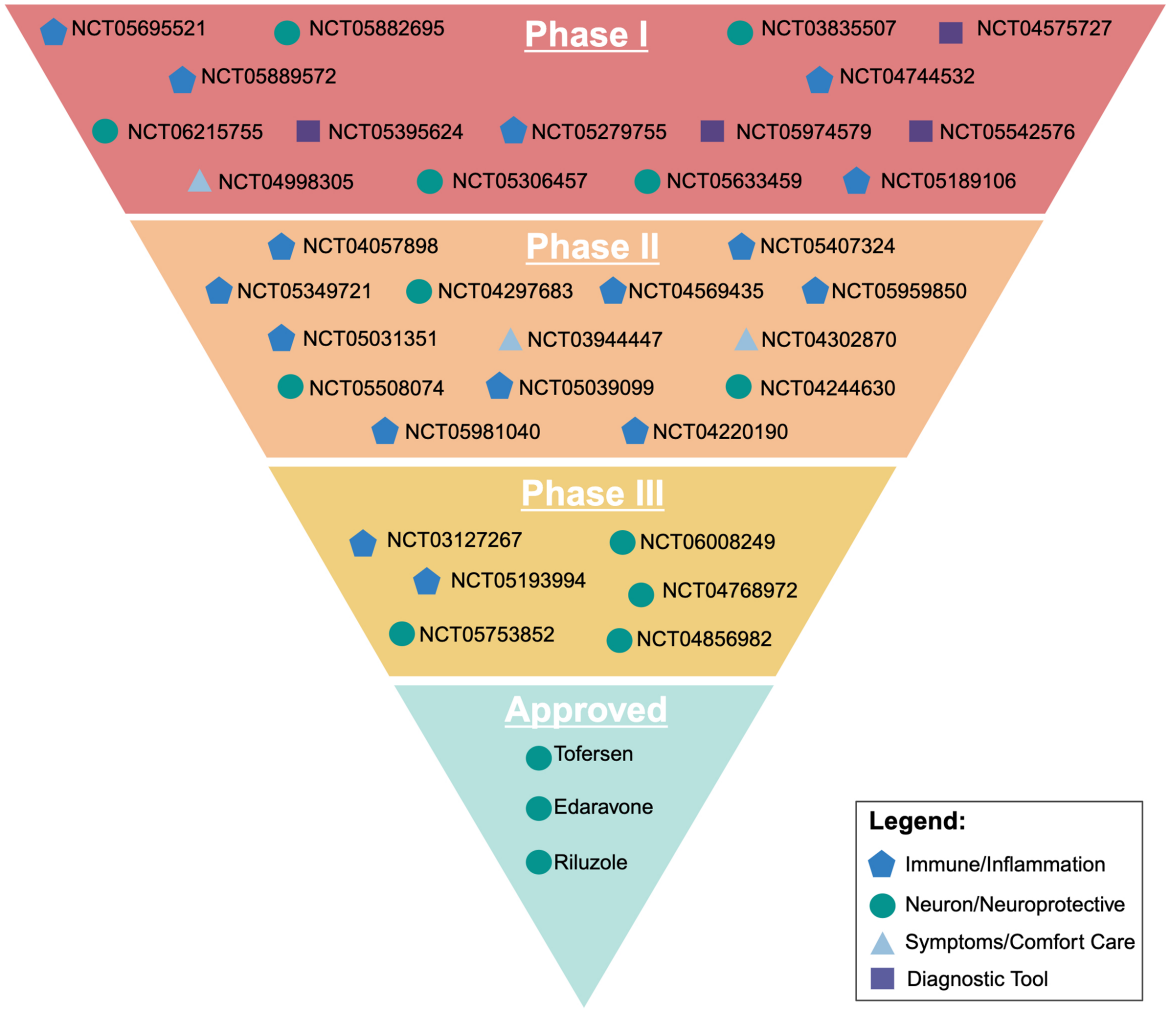
Visual representation of current ongoing clinical trials and their broad target of action outlined in the sponsors clinicaltrials.gov entry. Created with BioRender.com.

One way the ALS research community has tried to streamline clinical research is through the formation of the HEALY ALS Platform. According to the HEALEY ALS Platform Trial Research Partners,^1^ this master protocol platform first began enrolling patients in 2020, with the goal being to reduce the cost, speed up enrollment, and encourage more patient participation. This is accomplished by testing multiple treatments simultaneously, rather than one at a time. Currently, the HEALY Platform (Clinical Trials: NCT04297683) has drugs under Phase II/III with others in the pipeline. Below is a breakdown outlining the current on-going clinical trials with their broad mechanism we identified. These mechanisms are classified between targeting the immune system/neuroinflammation, the neuron/neuroprotective, symptoms/comfort care, or a diagnostic tool. It is important to reiterate that these pathways crosstalk and the true mechanism of the drug can be argued to be in multiple categories (Figure 2; Table 1).

**Table 1:** Table of all active clinical trials for ALS listed on clinicaltrials.gov.

NCT number	Sponsor	Title	Intervention/Drug Name	Study Phase	Broad effect
NCT04575727	Columbia University	Exploratory Evaluation of [11C]MPC6827	[11C]MPC6827	Early Phase 1	Diagnostic
NCT06215755	Verge Genomics	A Study of VRG50635 in Participants With Amyotrophic Lateral Sclerosis (ALS)	VRG50635	Phase 1	Neuron/Neuroprotection
NCT05882695	Spinogenix	Safety, Tolerability, Pharmacokinetics and Pharmacodynamics of SPG302 in Healthy Volunteers and ALS Participants	SPG302	Phase 1	Neuron/Neuroprotection
NCT05974579	Université de Sherbrooke	Safety and Dosimetry of a New Radiotracer to Detect Misfolded SOD1 Associated With Amyotrophic Lateral Sclerosis	89Zr-DFO-AP-101	Phase 1	Diagnostic
NCT05889572	MaaT Pharma	Safety and Gut Microbiota Analysis of an Oral Microbiotherapy in Patients With Amyotrophic Lateral Sclerosis	MaaT033	Phase 1	Immune/Inflammation
NCT05633459	QurAlis Corporation	A Study Evaluating the Safety and Tolerability of QRL-201 in ALS	QRL-201	Phase 1	Neuron/Neuroprotection
NCT05306457	Cedars-Sinai Medical Center	CNS10-NPC-GDNF Delivered to the Motor Cortex for ALS	CNS10-NPC-GDNF	Phase 1	Neuron/Neuroprotection
NCT05695521	Cellenkos, Inc.	Regulatory T Cells for Amyotrophic Lateral Sclerosis	CK0803	Phase 1	Immune/Inflammation
NCT05279755	ProJenX	A Study to Evaluate the Safety and Pharmacokinetics of Single and Multiple Doses of Prosetin in Healthy Volunteers	Prosetin	Phase 1	Immune/Inflammation
NCT04744532	Kyoto University	iPSC-based Drug Repurposing for ALS Medicine (iDReAM) Study	Bosutinib	Phase 1|Phase 2	Immune/Inflammation
NCT04998305	Hiroshi Mitsumoto	TJ-68 Clinical Trial in Patients With Amyotrophic Lateral Sclerosis (ALS) and Muscle Cramps	TJ-68	Phase 1| Phase 2	Symptom/Comfort Care
NCT03835507	Hanyang University Seoul Hospital	Randomized, Double-blind, Safety and Efficacy of Recombinant Human Erythropoietin in Amyotrophic Lateral Sclerosis	Recombinant human erythropoietin (rhEPO)	Phase 1| Phase 2	Neuron/Neuroprotection
NCT05189106	Massachusetts General Hospital	Neurodegenerative Alzheimer’s Disease and Amyotrophic Lateral Sclerosis (NADALS) Basket Trial	Baricitinib	Phase 1| Phase 2	Immune/Inflammation
NCT05542576	Amydis Inc.	AMDX-2011P Retinal Tracer in Subjects With Neurodegenerative Diseases Associated With Amyloidogenic Proteinopathy	AMDX2011P	Phase 1| Phase 2	Diagnostic
NCT05395624	Ashvattha Therapeutics, Inc.	Safety, PK and Biodistribution of 18F-OP-801 in Patients With ALS, AD, MS, PD and Healthy Volunteers	18F-OP-801	Phase 1| Phase 2	Diagnostic
NCT05407324	Corcept Therapeutics	Dazucorilant in Patients With Amyotrophic Lateral Sclerosis	Dazucorilant	Phase 2	Immune/Inflammation
NCT05349721	PTC Therapeutics	Study to Assess the Effects of PTC857 Treatment in Participants With Amyotrophic Lateral Sclerosis ALS	PTC857	Phase 2	Immune/Inflammation
NCT04569435	Annexon Biosciences	Study of ANX005 in Adults With Amyotrophic Lateral Sclerosis (ALS)	ANX005	Phase 2	Immune/Inflammation
NCT05508074	InFlectis BioScience	Treatment Combining Riluzole and IFB-088 in Bulbar Amyotrophic Lateral Sclerosis (TRIALS Protocol)	IFB-088	Phase 2	Neuron/Neuroprotection
NCT05039099	AL-S Pharma	A Study to Evaluate, Safety, Tolerability, Pharmacodynamic (PD) Markers and Pharmacokinetics (PK) of AP-101 in Participants With Amyotrophic Lateral Sclerosis (ALS)	AP-101	Phase 2	Immune/Inflammation
NCT05981040	Zydus Lifesciences Limited	Efficacy, Safety, Tolerability, Pharmacokinetics, and Pharmacodynamics of ZYIL1 in Patients With Amyotrophic Lateral Sclerosis	ZYIL1	Phase 2	Immune/Inflammation
NCT04244630	Dallas VA Medical Center	Mitochondrial Capacity Boost in ALS (MICABO-ALS) Trial	Antioxidants	Phase 2	Neuron/Neuroprotection
NCT05959850	The Florey Institute of Neuroscience and Mental Health	A Double-blind Randomised, Placebo-controlled Clinical Trial to Test Ambroxol Treatment in ALS	Ambroxol	Phase 2	Immune/Inflammation
NCT05031351	Sunnybrook Health Sciences Centre	NF-ĸB Inhibition in Amyotrophic Lateral Sclerosis	*Withania somnifera*	Phase 2	Immune/Inflammation
NCT03944447	OMNI Medical Services, LLC	Outcomes Mandate National Integration With Cannabis as Medicine	Cannabis	Phase 2	Symptom/Comfort Care
NCT04057898	MediciNova	Evaluation of MN-166 (Ibudilast) for 12 Months Followed by an Open-label Extension for 6 Months in Patients With ALS	MN-166	Phase 2| Phase 3	Immune/Inflammation
NCT04220190	Rapa Therapeutics LLC	RAPA-501 Therapy for ALS	RAPA-501 Autologous T cells	Phase 2| Phase 3	Immune/Inflammation
NCT04297683	Merit E. Cudkowicz, MD	HEALEY ALS Platform Trial – Master Protocol	ABBV-CLS-7262	Phase 2| Phase 3	Neuron/Neuroprotection
			DNL343		
NCT04302870	University of Edinburgh	Motor Neuron Disease – Systematic Multi-Arm Adaptive Randomised Trial	Memantine Hydrochloride Oral	Phase 2| Phase 3	Neuron/Neuroprotective
NCT05193994	Macquarie University, Australia	Triumeq in Amyotrophic Lateral Sclerosis	Dolutegravir, Abacavir and Lamivudine	Phase 3	Immune/Inflammation
NCT04768972	Ionis Pharmaceuticals, Inc	A Study to Evaluate the Efficacy, Safety, Pharmacokinetics and Pharmacodynamics of ION363 in Amyotrophic Lateral Sclerosis Participants With Fused in Sarcoma Mutations (FUS-ALS)	ION363	Phase 3	Neuron/Neuroprotection
NCT06008249	Stichting TRICALS Foundation	Platform Trial to Assess the Efficacy of Multiple Drugs in Amyotrophic Lateral Sclerosis (ALS)	Lithium Carbonate	Phase 3	Neuron/Neuroprotection
NCT03127267	AB Science	Efficacy and Safety of Masitinib Versus Placebo in the Treatment of ALS Patients	Masitinib	Phase 3	Immune/Inflammation
NCT05753852	Humanitas Mirasole SpA	Open Label Extension of TUDCA-ALS Study	Tauroursodeoxycholic Acid	Phase 3	Neuron/Neuroprotection
NCT04856982	Biogen	A Study of BIIB067 (Tofersen) Initiated in Clinically Presymptomatic Adults With a Confirmed Superoxide Dismutase 1 Mutation	Tofersen	Phase 3	Neuron/Neuroprotection

## 5 Discussion

Despite recent advancements in ALS research, significant gaps persist in our understanding of the disease’s pathophysiology, hindering the development of targeted therapies. While precision medicine has made strides, like with the development of Tofersen for *SOD1* mutant patients, the fundamental triggers initiating ALS pathology remain elusive. Common neurodegenerative disease pathways involving mitochondrial dysfunction, neuroinflammation, and glutamate-mediated cytotoxicity have been identified, however, the primary driving force being disease progression remains elusive.

One critical aspect that demands attention is the need for a more robust diagnostic pipeline. The prolonged delay in ALS diagnosis, which in some cases can take up to 14 months, poses substantial challenges for patient outcomes. An improved and expedited diagnostic process would allow for earlier treatment intervention, potentially leading to better survival outcomes and allowing patients to maintain their independence longer. A comprehensive understanding of the heterogeneity within ALS, both genetic and non-genetic factors, is essential for tailoring treatment to the individual patient.

Another significant hurdle lies in drug development. Targeting a single mechanism has proven to be insufficient, with the majority of clinical trials failing to meet primary endpoints. This may be attributed to the potential circumvention of certain pathways, enabling neurodegeneration to persist. While a universal approach would be ideal, acknowledging ALS as a heterogeneous disease suggests the need for a more nuanced approach. Stratifying patients based on their underlying pathology and genetic mutations may lead to more efficacious clinical trial designs.

The emergence of the HEALY ALS Platform spotlights the efforts made to streamline clinical research and accelerating drug development. However, the dynamic and reactive nature of ALS pathology, as highlighted in this review, underscores the complexity that may impede the success of monotherapeutic approaches. Collaborative efforts should be directed toward understanding the intricacies present and identifying synergistic interventions. Ultimately, ALS remains a challenging neurodegenerative disease to understand. Despite the continued progress, the factors contributing to disease progression needs continued research and collaboration. By addressing the gaps in our knowledge and optimizing both therapeutic development and diagnostic tools, there is hope for improving outcomes and the quality of life for individuals living with ALS.

## Author contributions

DB: Writing – original draft, Writing – review & editing. KR: Writing – original draft, Writing – review & editing.

## Footnotes

^1^http://massgeneral.org
